# Preliminary Validation of the Revised Illness Perception Questionnaire for Patients with Nasopharyngeal Carcinoma in China

**DOI:** 10.3390/healthcare11182469

**Published:** 2023-09-05

**Authors:** Yuqi Cai, Yuan Zhang, Wangnan Cao, Vivian Yawei Guo, Yang Deng, Liying Luo, Jianling Shen, Yang Zhu, Xiaoting Chen, Xiao Yang, Fengsu Hou, Jinghua Li

**Affiliations:** 1 School of Public Health, Sun Yat-Sen University, Guangzhou 510080, Chinalijinghua3@mail.sysu.edu.cn (J.L.); 2 Department of Radiation Oncology, Sun Yat-Sen University Cancer Center, State Key Laboratory of Oncology in South China, Collaborative Innovation Center for Cancer Medicine, Guangdong Key Laboratory of Nasopharyngeal Carcinoma Diagnosis and Therapy, Guangzhou 510080, China; 3 Department of Social Medicine and Health Education, School of Public Health, Peking University, Beijing 100871, China; 4 Shenzhen Kangning Hospital/Shenzhen Mental Health Center, Shenzhen 518020, China

**Keywords:** nasopharyngeal carcinoma, illness perception, validation

## Abstract

Nasopharyngeal carcinoma is a common and highly malignant cancer in southern China. It is important to accurately assess the illness perception of nasopharyngeal carcinoma according to the common-sense model of self-regulation. The purpose was to validate the Chinese version of the Revised Illness Perception Questionnaire for patients with Nasopharyngeal carcinoma. A cross-sectional survey of 631 patients with Nasopharyngeal carcinoma was conducted in Guangzhou, China. The reliability of the scale was evaluated using Cronbach’s alpha. The factor structure was assessed using exploratory factor analysis (EFA) of each dimension. The EFA revealed that the 29-item self-rated scale has a seven-factor structure consistent with the original scale and explained 67.3% of the variance after extraction and rotation. The scale showed satisfactory reliability. The item–total correlations ranged from −0.16 to 0.64 (*p* < 0.05). The item–subscale correlations ranged from 0.46 to 0.91 (*p* < 0.05). The item–other subscale correlations ranged from −0.38 to 0.51 and from −0.21 to 0.56 (*p* < 0.05). Significant correlations were found between the timeline (acute/chronic) (*r* = 0.224, *r* = 0.166), consequences (*r* = 0.415, *r* = 0.338), timeline cyclical (*r* = 0.366, *r* = 0.284), emotional representations (*r* = 0.497, *r* = 0.465), personal control (*r* = −0.122, *r* = −0.134), treatment control (*r* = −0.135, *r* = −0.148), and illness coherence (*r* = −0.261, *r* = −0.213) subscales, and depression, anxiety (*p* < 0.05). The scale revealed acceptable reliability, factorial validity, and construct validity. It could be used to assess the illness representations of Chinese patients with nasopharyngeal carcinoma.

## 1. Introduction

According to the International Agency for Research on Cancer, there were 129,079 new cases of nasopharyngeal carcinoma (NPC) worldwide in 2018 [1], among which 70% were concentrated in east and southeast Asia [1,2]. China’s age-standardized NPC incidence rate was about eight times higher than that of regions with predominantly white populations in 2018 [1,2]. Progress in clinical treatment has remarkably reduced NPC mortality [3] and improved overall survival [4].

NPC patients are at high risk of psychological distress [5] and poor quality of life [6] due to the stigmatizing nature of the facial changes caused by the disease [7], and debilitating therapy, which interfere with social function, speech, eating, cognitive function, and sexual function [8,9]. The incidence of depression and anxiety among NPC patients at the end of radiotherapy is approximately 56% and 64%, respectively [10]. Poor mental health may lead to undesired behaviors [11], including poor adherence to treatment and self-management [12], which further affect physical and mental health [13] and quality of life [14], creating a vicious cycle.

According to the common-sense model of self-regulation [15,16], patients’ illness representation or perception plays an important role in self-regulation. The model suggests that cognitive and emotional elements of illness representations are significantly associated with the coping behaviors, mental health status, and quality of life of patients with hypertension [11], diabetes [17], and cancers, such as chronic lymphocytic leukemia [18], gastrointestinal cancer [19], colorectal cancer [20], and breast cancer [21]. Studies have shown that illness perceptions are associated with depression and anxiety [22]. The consequence dimension of illness perception was shown to be positively correlated with anxiety (*r* = 0.23, *p* < 0.05) and depression (*r* = 0.27, *p* < 0.05), while the illness coherence dimension was negatively correlated with anxiety (*r* = −0.47, *p* < 0.05) and depression (*r* = −0.26, *p* < 0.05) [23]. Thus, depression and anxiety were used to test criterion-related validity in this study.

The Illness Perception Questionnaire (IPQ) is the most widely used measurement of illness perception. It is based on Kleinman’s explanatory model of illness and provides a comprehensive explanation of patient perceptions of the diagnosis, treatment, course, cause, and nature of a given disease, which combine to construct their beliefs and understandings from a life course perspective [24]. The Illness Perception Questionnaire-Revised (IPQ-R) was developed to comprehensively assess six dimensions of cognitive representations and one dimension of emotional representations of illness [24,25]. The cognitive dimensions of the IPQ-R include timeline (acute/chronic), consequences, personal control, treatment control, illness coherence, and timeline cyclical. It has been translated into and validated in different languages worldwide for patients with various chronic diseases and has shown good reliability and validity [24].

Patients with different types of cancer may hold different perceptions, beliefs, and experiences. However, there is a lack of literature on the personal experiences of NPC patients. In addition, although a few studies have validated the IPQ-R for patients with cancer, such as breast cancer [26], most were conducted in Western populations [24]. Furthermore, the psychometric properties of the IPQ-R among patients with NPC have not been broadly examined. We identified only four studies that tested the IPQ-R among Chinese patients, specifically those with breast cancer-related lymphedema [27], breast cancer [26], and cervical cancer [28].

The illness perception among cancer patients is related to their cultural background [29]. For example, yin and yang, as a concept in Taoism, has deeply influenced Chinese culture and health beliefs [30]. Traditional Chinese medicine believes that health cannot be achieved without a balance of yin and yang [30]. People in the Chinese culture often believe that the reason they can control the disease is mainly because they fully understand the disease [31]. Furthermore, in China, the distribution of NPC shows obvious geographical differences, with a higher prevalence in southern regions, especially Cantonese-speaking regions Guangdong than in the north [32]. People living in high-risk areas may have a stronger perception of disease risk, in addition to the fact that Guangdong leads the nation in treating NPC. These geographic factors may also influence the illness perception. Thus, given that Chinese NPC patients may have different illness perceptions due to the cultural context and disease-specific characteristics, this study aimed to identify how NPC patients experience the disease and to develop and validate the Chinese version of the Illness Perception Questionnaire Revision for patients with Nasopharyngeal Carcinoma (R-NPC-IPQ) based on the English version of the IPQ-R. We anticipated that the study would shed light on NPC patients’ experiences and thus improve the quality of health services.

## 2. Materials and Methods

### 2.1. Participants

A cross-sectional survey was conducted at Sun Yat-sen University Cancer Center (Guangzhou, China). The inclusion criteria were (1) 18 years of age or older, (2) histologically documented diagnosis of NPC, (3) have not ever been diagnosed with any psychiatric disorders, (4) appropriate knowledge of the Chinese language and satisfactory level of communication, and (5) consent to participate in the study. The exclusion criteria included (1) the presence of serious tumors other than NPC, (2) intellectual disability, and (3) inability to complete the questionnaire independently.

### 2.2. Data Collection

**Figure 1:**
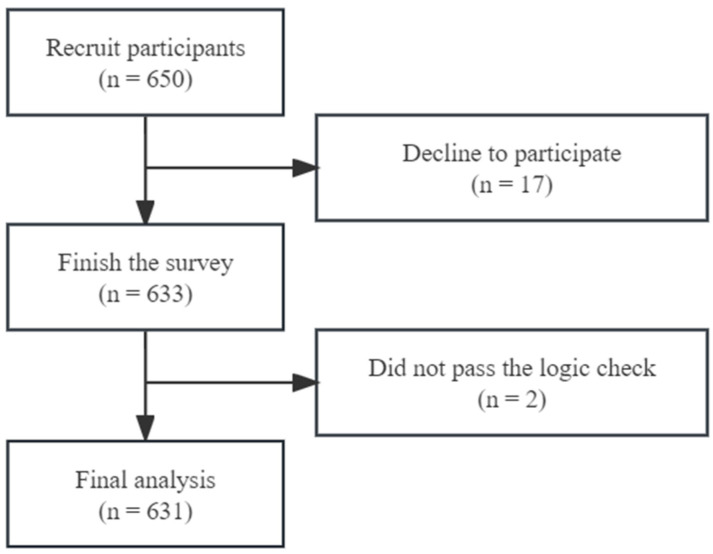
Flow chart of recruitment.

Between patients waiting for their appointments or waiting for radiation therapy, trained research assistants identified and approached eligible patients while distributing the study materials in the outpatient clinic. Investigators briefly introduced himself/herself as the investigator and provided study details (including the purpose of the study, the study sponsoring organization, and the main content of the study) to the patients and asked them if they would like to participate in this survey. The participants were assured that refusal would not affect their medical services at the hospital, and they could quit at any time during the survey without being questioned. If the patient is willing to participate, they will sign a paper consent form prior to the survey. A total of 650 patients were approached, of whom 17 declined to participate, and 633 (response rate: 97.3%) completed the survey. Of the 633 questionnaires collected, two did not pass the logic check, and the remaining 631 participants were included (Figure 1). Ethical approval was obtained from the Ethics Committee of Sun Yat-sen University (No. 2019-145).

### 2.3. Measures

#### 2.3.1. Development of the Chinese Version of the R-NPC-IPQ

The process of translating and culturally adapting the scale includes the following steps [33]: (1) experts in NPC and psychology reviewed and discussed the item pool, (2) two translators translated the scale into Chinese, (3) experts fluent in both Chinese and English were invited to validate the translation and adapt it to the cultural context of China, (4) we did back translation and compare the back-translated versions of the scale with the original, (5) a draft version of the scale was pretested on 19 patients.

The final version of the R-NPC-IPQ is a 38-item scale containing seven dimensions that evaluate the perceptions and counter-perceptions resulting from the process or experience of coping with cancer: (1) timeline chronic–acute (perceived degree of chronicity of the disease; 6 items), (2) consequences (consequences of the disease for different areas of the patient’s life; 6 items), (3) personal control (perceived ability to control the disease; 6 items), (4) treatment control (perception of the extent to which treatment can control the disease; 5 items), (5) disease coherence (general understanding of the disease; 5 items), (6) timeline periodicity (perceived course of the disease from the periodic appearance of symptoms; 4 items), and (7) emotional representations (emotional burden caused by the disease; 6 items) [34]. Among them, items 1, 4, 8, 15, 17, 18, 19, 23, 24, 25, 26, 27, and 36 are reverse scored. The participants were instructed to score the items on a 5-point Likert scale (1 = strongly disagree, 2 = disagree, 3 = neither agree nor disagree, 4 = agree, 5 = strongly agree). The total scores ranged from 38 to 190, and higher scores represented higher levels of the relevant dimension. For example, high scores on the emotional representation items indicated high levels of emotional stress.

#### 2.3.2. Generalized Anxiety Disorder 7 (GAD-7)

The Generalized Anxiety Disorder 7 (GAD-7) consists of seven items. It is used primarily to identify potential generalized anxiety and assess its degree. The scale has been widely used in China, with good content and construct validity reported [35]. A 4-point Likert-like scale is used to measure self-reported anxiety levels from 0 (almost never) to 3 (almost every day), with total scores ranging from 0 to 21. The total scores of 0–4, 5–9, 10–14, and 15–21 are often used to classify increasing severity of anxiety. A score of 10 or above indicates anxiety disorder [36]. The Cronbach’s alpha was 0.947 in this study. Higher scores indicate higher levels of anxiety. The total score of this scale will be analyzed by Pearson correlation with the total IPQ score and the scores of each dimension.

#### 2.3.3. Patient Health Questionnaire (PHQ-9)

The Patient Health Questionnaire (PHQ-9) is a 9-item self-administered diagnostic tool to identify the presence of depression. Participants rate their depressive symptoms in the previous two weeks on a 4-point Likert-like scale, ranging from 0 (not at all) to 3 (almost every day). The total score ranges from 0 to 27. The total summed scores of 0–4, 5–9, 10–14, 15–19, and 20–27 are often used to classify non-mild, mild, moderate, moderate-severe, and severe depression, respectively. A score of 10 or above was classified as a depressive disorder [37]. The Chinese version of the PHQ-9 has good psychometric properties among Chinese cancer populations [38]. The Cronbach’s alpha value was 0.915 in this study. Higher scores indicate higher levels of depression. The total score of this scale will be analyzed by Pearson correlation with the total IPQ score and the scores of each dimension.

#### 2.3.4. Demographic Information

Socio-demographic data were collected, including age, gender, education, marital status, occupation, household economic status, and health care payment patterns.

### 2.4. Statistical Analysis

The data were analyzed using IBM SPSS version 25.0 (IBM Corp., Armonk, NY, USA). All statistical tests were two-sided, and the significance level was set at 0.05. Frequencies, percentages, means, and standard deviations were used for the descriptive analysis. The construct validity of the R-NPC-IPQ was assessed using exploratory factor analysis (EFA) based on a 7-factor model. We conducted Kaiser–Meyer–Olkin (KMO) and Bartlett’s sphericity tests to measure the sampling adequacy and appropriateness of the factor analysis [39].

The factor structure of the IPQ-R was assessed by EFA based on principal component analysis (PCA) with oblique rotation. The factor contributions were assessed using the Kaiser criterion with a cut-off eigenvalue higher than 1.0 and visual inspection of the scree plot. We used breakpoints in the scree plot to determine which figure provided the cleanest and most interpretable structure. Contributing items were identified if their factor loadings were greater than 0.4. To further improve the interpretability of the structure, items with weak loadings (≤0.4) were removed. Any cross-loaded items were assigned to the factor they best fit in terms of conceptual similarity. The internal reliability of the scale was evaluated using Cronbach’s alpha coefficients [39,40].

Pearson correlation analysis was applied to compare item–total correlations, item–subscale correlations, and item–other-subscale correlations within different R-NPC-IPQ subscales. A specific item was properly classified into the current corresponding subscale rather than other subscales if the item–subscale correlations were higher than the item–other-subscale correlations. In addition, the external validity of the R-NPC-IPQ was assessed by examining the Pearson correlation coefficients between the R-NPC-IPQ and two external variables (i.e., GAD-7 and PHQ-9 scores).

## 3. Results

### 3.1. Descriptive Statistics

The demographic characteristics of the participants are summarized in Table 1. The participants were aged 18 to 88 years, with a mean of 46.9 years (SD = 11.3 years). Of the 631 participants, 69.1% were male; the majority were married (89.9%); 97.3% were Han Chinese; 59.4% were urban residents; 43.6% were unemployed; 32.3% had an average monthly household income of 3001–5000 RMB; and 31.7% had completed university education. For insurance, 98.6% had medical insurance, and 43.9% had borrowed money due to illness (Table 1).

The mean score of the R-NPC-IPQ was 91.30 (SD = 11.25). The percentage of responses affected by the floor effect for all items ranged from 1.6% to 22.0%. The percentages of responses affected by the ceiling effect ranged from 2.1% to 22.7% (Table 2).

The prevalence of probable depression and anxiety was 45.6% and 35.2%, respectively. The mean scores of the GHQ-9 and GAD-7 were 5.30 (SD = 5.54) and 3.65 (SD = 4.43), respectively.

### 3.2. Factor Structure

#### 3.2.1. Exploratory Factor Analysis

The results of the Kaiser–Meyer–Olkin (KMO) (0.875) and Bartlett’s tests (χ^2^ = 9489.501, df = 406, *p* < 0.001) indicated good factor analysis performance. In the EFA, we identified seven significant factors with eigenvalues greater than 1, which was consistent with the original IPQ-R scale. We removed nine items and re-conducted the PCA with oblique rotation on the remaining 29 items, yielding seven factors. Before the EFA, items 17 and 19 were deleted as they duplicated the meaning of item 16 and item 23, respectively. This improved the overall value of Cronbach’s alpha from 0.718 to 0.740. In the assessment of item contributions, there were five items with factor loadings of 0.4 or below. These were item 1, item 15, item 18, item 23, and item 36. We removed two items (items 4 and 6) that belonged to more than one dimension, The eigenvalues for the seven factors were 7.74, 3.46, 2.33, 1.94, 1.68, 1.28, and 1.11, respectively, explaining 26.7%, 11.9%, 8.0%, 6.7%, 5.8%, 4.4%, and 3.8% of the variance, which together explained 67.3% of the total variance. The factor loadings and contributions of each item are summarized in Table 3.

#### 3.2.2. Item Analysis and Reliability

The Cronbach’s alpha coefficients ranged from 0.73 to 0.92 for the seven dimensions of the scale, which indicates good internal consistency (Table 3). The item–total correlation coefficients ranged from −0.16 to 0.64 (*p* < 0.05, Table 4). The item–subscale correlation coefficients ranged from 0.46 to 0.91 (*p* < 0.05, Table 4). Moderately strong correlations were detected between the subscales. The item–other-subscale correlation coefficients ranged from −0.38 to 0.56 (*p* < 0.05, Table 4). The mean total scores of the seven dimensions were 7.82 (SD = 3.03), 15.20 (SD =3.89), 13.02 (SD = 2.99), 11.07 (SD = 2.13), 15.77 (SD = 3.77), 9.93 (SD = 3.24), and 13.95 (SD = 4.58), respectively (Table 5). 

#### 3.2.3. External Correlation

Regarding external validity (Table 6), chronic timeline, consequences, higher timeline cycle, and emotional representations showed mild to moderate positive correlations with depression (r = 0.224, 0.415, 0.366, and 0.497, respectively, *p* < 0.05) and anxiety (r = 0.166, 0.338, 0.284, and 0.465, respectively, *p* < 0.05), while personal control, treatment control, and illness coherence showed mild negative correlations with depression (r = −0.122, −0.135, −0.261, respectively, *p* < 0.05) and anxiety (r = −0.134, −0.148, −0.213, respectively, *p* < 0.05). The external correlations with depression and anxiety are summarized in Table 6.

## 4. Discussion

We conducted EFA on data from 631 patients with NPC to validate the structural and content validity of the R-NPC-IPQ. The results confirmed that the 29-item R-NPC-IPQ possessed satisfactory factorial validity, external validity, and internal reliability. This is the first study to investigate illness representation and IPQ-R use among NPC patients in China and to report reliable results. The GAD-7 and PHQ-9 showed good reliability in this study, which similarly confirmed the high prevalence of anxiety and depression in patients with NPC.

The removal of items 1, 4, 6, 15, 17, 18, 19, 23, and 36 would improve the structural validity of the IPQ-R given the weak factor loadings and cross factor loadings, a finding similar to IPQ-R validation studies among patients with Internet gaming disorder [41], type 2 diabetes [42], and cervical cancer [28]. Most of the removed items were reverse-scored. Other researchers have encountered similar problems and eventually removed items with reversed wording. Although reversed items are commonly used to prevent response biases, researchers have pointed out that they may weaken scale validity because the responses to reversed items are more prone to errors due to inattention and confusion [43]. Cultural differences may also contribute to the poor performance of the removed items. For instance, item 18 from the timeline chronic factor, “My NPC will improve in time,” is difficult to understand and ambiguous in different cultural contexts. In Chinese culture, patients often adapt to health threats by adopting strategies such as “seeking bliss” and “letting fate take its course”, believing that agreeing with negative aspects will lead to correspondingly undesirable outcomes [43]. Thus, they may interpret statements such as item 15, “Nothing I do will affect my NPC”, and item 23, “There is nothing that can help my condition” as unwanted fatalism and intentionally underscore these items while intentionally overscoring those items conveying positive expectations such as item 18, “My NPC will improve in time”, and item 36, “My NPC does not worry me”.

Patients with NPC are exposed to severe stigma and several types of stressors. It was not surprising that we found a high prevalence of probable depression and anxiety in this study sample, which was consistent with studies conducted in Western countries [44]. Positive correlations were found between mental health problems and the consequences, illness coherence, timeline cycle, and emotional representation factors. This finding was supported by the common-sense model of self-regulation, which posits that cognitive and emotional representations play an important role in self-regulation among patients facing health threats, which further affects their mental health and quality of life. Studies have also found that these factors were positively associated with depression and anxiety among patients with breast cancer [45], type 2 diabetes [46], and head and neck cancer [47]. The findings suggest that reducing misunderstanding and improving knowledge about NPC and its treatments may reduce mental health problems. The personal control factor was negatively associated with depression, suggesting that a higher level of perceived personal control is a protective factor. Interventions are warranted to increase patients’ self-efficacy in managing their illness.

The 29-item modified R-NPC-IPQ showed satisfactory psychometric properties compared with the original IPQ-R in terms of internal consistency, factorial validity, and external validity. The Cronbach’s alphas of the modified scale ranged from 0.73 to 0.92, higher than that of the original IPQ-R. Factor analysis yielded seven significant factors with satisfactory eigenvalues, which was consistent with the original IPQ-R scales and other IPQ-R validation studies among cancer patients, such as those with breast cancer. The mild to moderate inter-factor correlations confirmed that each factor measured a distinct construct of illness representation. External validity was supported by the significant positive correlations of depression and anxiety with the factors for chronic timeline, consequences, higher timeline cycle, and emotional representations, and their negative correlations with personal control, treatment control, and illness coherence.

This study is the first to validate the IPQ-R with a relatively large sample of patients with NPC. However, it has several limitations. First, our sample was recruited from an outpatient clinic. Thus, the results cannot be generalized to hospitalized patients with NPC due to the difference in clinical severity. Further validation studies are greatly warranted to test the factor structure of the IPQ with NPC inpatients. Second, the findings may not be generalizable to Western populations due to cultural differences. Third, causal inferences cannot be made as we used a cross-sectional study design. Fourth, test–retest reliability was not validated in this study. Furthermore, confirmatory factor analysis (CFA) and test-retest reliability analyses were not performed in this study. We hope that future studies using this scale will conduct further analyses.

## 5. Conclusions

In conclusion, the modified 29-item R-NPC-IPQ has acceptable reliability and validity and is suitable for assessing the illness representations of Chinese patients with NPC. This study explored the associations between cognitive and emotional perceptions with mental health outcomes. Using this validated scale, future research is warranted to examine the mechanism by which illness representations of NPC influence patients’ behaviors and then further affect their mental health and quality of life. Studies to develop and test the efficacy of interventions to modify illness representations are also warranted.

## Figures and Tables

**Table 1:** Demographic characteristics of the samples with NPC (*n* = 631).

Socio-Demographic Variables	Levels	*n* (%)
Age	<40 years old	179 (28.4)
	40–59 years old	370 (58.6)
	60–85 years old	82 (13.0)
Sex	Male	436 (69.1)
	Female	195 (30.9)
Ethnicity	Han Chinese	614 (97.3)
	Other	17 (2.7)
Residence	Urban	375 (59.4)
	Rural	256 (40.6)
Marriage	Currently Unmarried	64 (10.1)
	Currently Married	567 (89.9)
Primary Caregiver	Spouse	443 (70.2)
	Children	99 (15.7)
	Parent and Siblings	65 (10.3)
	Myself	24 (3.8)
Education	Junior High School and below	287 (45.5)
	High School	144 (22.8)
	University and above	200 (31.7)
Occupation	Full-time and Part-time	279 (44.2)
	Retired	77 (12.2)
	Unemployed	223 (35.3)
	Other	52 (8.2)
Average Monthly Income	Less than 3000 RMB	164 (26.0)
	3001–5000 RMB	204 (32.3)
	5001–10,000 RMB	160 (25.4)
	Above 10,000 RMB	103 (16.3)
Type of Medical Insurance	Urban Employee-Based Medical Insurance	243 (38.5)
	Urban Resident-Based Medical Insurance	164 (26.0)
	New Cooperative Medical Scheme	215 (34.1)
	No Medical Insurance	9 (1.4)
Borrowing Money for Illness	Yes	277 (43.9)
	No	354 (56.1)

**Table 2:** Descriptive statistics for the items in the R-NPC-IPQ (*N* = 631).

Subscales	Items	Score Range	Mean ^1^	SD ^2^	Floor Effect ^3^ (%)	Ceiling Effect ^3^ (%)	Skewness ^4^	Kurtosis ^4^
Timing (acute/chronic)	1. My NPC will last a short time	1~5	2.68	1.141	14.3	9.7	0.474	−0.418
	2. My NPC is likely to be permanent rather than temporary	1~5	2.63	1.130	19.5	5.1	0.159	−0.762
	3. My NPC will last for a long time	1~5	2.68	1.104	17.0	4.3	0.093	−0.792
	4. This NPC will pass quickly	1~5	2.57	1.061	14.7	6.8	0.535	−0.063
	5. I expect to have this NPC for the rest of my life	1~5	2.51	1.128	22.0	4.8	0.328	−0.678
	18. My NPC will improve in time	1~5	2.51	0.939	10.0	4.4	0.719	0.473
Consequences	6. My NPC is a serious condition	1~5	2.53	1.105	20.0	4.0	2.53	1.105
	7. My NPC has major consequences on my life	1~5	2.82	1.139	14.3	6.0	2.82	1.139
	8. My NPC does not have much effect on my life	1~5	3.23	1.134	7.3	13.3	3.23	1.134
	9. My NPC strongly affects the way others see me	1~5	2.73	1.083	15.8	4.1	2.73	1.083
	10. My NPC has serious financial consequences	1~5	3.45	1.133	7.1	17.1	3.45	1.133
	11. My NPC causes difficulties for those who are close to me	1~5	2.98	1.097	10.6	6.0	2.98	1.097
Personal Control	12. There is a lot I can do to control my symptoms	1~5	3.16	0.971	6.7	5.9	−0.380	−0.115
	13. What I do can determine whether my NPC gets better or worse	1~5	3.39	0.967	4.9	8.7	−0.585	0.072
	14. The course of my NPC depends on me	1~5	3.27	0.954	5.1	6.0	−0.502	−0.111
	15. Nothing I do will affect my NPC	1~5	3.58	0.987	2.5	17.7	−0.417	−0.259
	16. I have the power to influence my NPC	1~5	3.20	1.009	6.7	6.7	−0.408	−0.336
	17. My actions will have no effect on the outcome of my NPC	1~5	3.49	0.959	1.9	13.8	−0.292	−0.415
Treatment Control	19. There is very little that can be done to improve my NPC	1~5	3.26	1.016	4.0	12.5	−0.057	−0.452
	20. My treatment will be effective in curing my NPC	1~5	3.73	0.827	1.9	14.6	−0.719	1.066
	21. The negative effects of my NPC can be prevented (avoided) by my treatment	1~5	3.62	0.832	2.1	11.3	−0.621	0.798
	22. My treatment can control my NPC	1~5	3.72	0.839	2.4	12.5	−0.949	1.406
	23. There is nothing that can help my condition	1~5	3.76	0.949	1.6	22.7	−0.547	−0.082
Illness Coherence	24. The symptoms of my condition are puzzling to me	1~5	2.99	1.009	4.1	8.7	0.299	−0.501
	25. My NPC is a mystery to me	1~5	3.17	1.042	4.0	11.6	0.065	−0.663
	26. I don’t understand my NPC	1~5	3.23	1.013	2.9	11.3	0.022	−0.682
	27. My NPC doesn’t make any sense to me	1~5	3.19	1.009	3.8	10.1	−0.006	−0.557
	28. I have a clear picture or understanding of my condition	1~5	3.20	0.962	5.9	5.9	−0.408	−0.135
Timeline Cyclical	29. The symptoms of my NPC change a great deal from day to day	1~5	2.66	0.992	13.0	2.9	0.101	−0.476
	30. My symptoms come and go in cycles	1~5	2.37	0.969	18.1	2.2	0.488	−0.154
	31. My NPC is very unpredictable	1~5	2.53	0.982	15.2	2.4	0.240	−0.417
	32. I go through cycles in which my NPC gets better and worse.	1~5	2.38	0.966	18.2	2.1	0.430	−0.227
Emotional Representation	33. I get depressed when I think about my NPC	1~5	2.72	1.065	13.5	3.6	0.084	−0.782
	34. When I think about my NPC, I get upset	1~5	2.73	1.071	13.3	4.6	0.132	−0.689
	35. My NPC makes me feel angry	1~5	2.72	1.028	11.9	3.8	0.123	−0.595
	36. My NPC does not worry me	1~5	3.11	1.063	6.3	10.3	−0.024	−0.588
	37. Having this NPC makes me feel anxious	1~5	2.92	1.051	9.8	4.4	−0.121	−0.749
	38. My NPC makes me feel afraid	1~5	2.85	1.065	11.7	4.6	−0.063	−0.716

^1^ Mean, simple arithmetic mean, describes the centralized trend of item scores; ^2^ SD, standard deviation, describes the dispersion trend of item scores. ^3^ Floor Effect (%) and Ceiling Effect (%) describe the sensitivity of questionnaire items to experimenters under such experimental conditions. ^4^ Skewness and Kurtosis describe the symmetry and steepness of item scores distribution.

**Table 3:** Exploratory factor analysis of the R-NPC-IPQ items and factor loadings of the final model.

Subscales	Items	Factor 1	Factor 2	Factor 3	Factor 4	Factor 5	Factor 6	Factor 7
Emotional Representation	37. Having this NPC makes me feel anxious	**0.907**	0.029	−0.059	0.059	−0.023	0.043	0.016
	34. When I think about my NPC I get upset	**0.886**	−0.042	−0.026	−0.035	0.049	−0.006	0.010
	38. My NPC makes me feel afraid	**0.856**	0.006	0.014	0.019	0.009	0.019	0.004
	35. My NPC makes me feel angry	**0.843**	0.035	0.017	−0.073	−0.038	−0.067	−0.013
	33. I get depressed when I think about my NPC	**0.760**	−0.024	0.034	−0.016	0.087	0.083	−0.007
Personal Control	13. What I do can determine whether my NPC gets better or worse	0.023	**0.830**	0.041	0.002	0.011	0.011	0.011
	14. The course of my NPC depends on me	−0.053	**0.812**	0.066	−0.027	−0.010	0.138	−0.044
	16. I have the power to influence my NPC	0.065	**0.751**	−0.088	−0.033	−0.028	−0.122	0.000
	12. There is a lot I can do to control my symptoms	−0.018	**0.663**	−0.019	0.012	−0.052	0.021	0.033
Timeline (acute/chronic)	2. My NPC is likely to be permanent rather than temporary	0.002	−0.052	**0.903**	−0.032	−0.043	−0.049	−0.008
	3. My NPC will last for a long time	−0.011	0.044	**0.880**	−0.009	0.005	0.013	−0.075
	5. I expect to have this NPC for the rest of my life	−0.012	0.023	**0.862**	−0.020	0.038	0.009	−0.108
Illness Coherence	26. I don’t understand my NPC	−0.008	0.033	−0.073	**0.863**	−0.046	−0.033	−0.029
	27. My NPC doesn’t make any sense to me	−0.020	−0.036	0.004	**0.849**	−0.016	0.023	0.012
	25. My NPC is a mystery to me	−0.064	−0.019	−0.026	**0.810**	−0.093	0.008	−0.101
	24. The symptoms of my condition are puzzling to me	−0.034	−0.080	−0.044	**0.654**	−0.166	−0.035	−0.048
	28. I have a clear picture or understanding of my condition	0.001	0.157	0.135	**0.458**	0.376	−0.067	0.302
Timeline Cyclical	29. The symptoms of my NPC change a great deal from day to day	−0.072	−0.013	−0.080	−0.019	**0.802**	0.079	0.017
	30. My symptoms come and go in cycles	0.098	−0.061	0.016	−0.079	**0.764**	0.014	−0.087
	31. My NPC is very unpredictable	0.090	−0.033	0.068	−0.179	**0.721**	0.021	−0.089
	32. I go through cycles in which my NPC gets better and worse.	0.267	−0.097	0.073	−0.103	**0.634**	−0.033	−0.113
Consequences	11. My NPC causes difficulties for those who are close to me	0.072	0.142	0.015	−0.051	0.001	**0.752**	0.022
	10. My NPC has serious financial consequences	−0.036	0.032	−0.016	−0.115	0.107	**0.733**	0.123
	8. My NPC does not have much effect on my life	0.067	−0.045	−0.112	0.102	0.029	**0.583**	−0.257
	7. My NPC has major consequences on my life	0.139	−0.088	0.311	0.001	−0.020	**0.537**	0.044
	9. My NPC strongly affects the way others see me	0.169	−0.099	0.328	−0.027	−0.054	**0.432**	0.106
Treatment Control	20. My treatment will be effective in curing my NPC	0.039	0.014	−0.083	−0.015	−0.079	−0.087	**0.835**
	22. My treatment can control my NPC	−0.020	0.010	−0.050	−0.008	0.022	0.030	**0.830**
	21. The negative effects of my NPC can be prevented (avoided) by my treatment	−0.019	0.044	−0.061	−0.049	−0.073	0.070	**0.786**
Cronbach’s Alpha		0.917	0.768	0.884	0.803	0.847	0.734	0.809
Initial Eigenvalues		7.739	3.455	2.332	1.937	1.677	1.279	1.112
Cumulative % of variance explained		26.686	38.599	46.640	53.320	59.104	63.512	67.346

Note: Extraction Method: Principal Component Analysis. Rotation Method: Direct Oblimin with Kaiser Normalization. Bolded content indicates loadings greater than 0.4.

**Table 4:** Item analysis of the of the R-NPC-IPQ.

Subscales	Items	Cronbach’s Alpha if Item is Deleted		Item-Total Correlation	Item-Subscale Correlation	Item-Other-Subscale Correlation
		Subscale	Total			
Timeline (acute/chronic)	2. My NPC is likely to be permanent rather than temporary	0.835	0.724	0.444 **	0.902 **	−0.182 **–0.325 **
	3. My NPC will last for a long time	0.830	0.720	0.499 **	0.902 **	−0.054–0.383 **
	5. I expect to have this NPC for the rest of my life	0.841	0.721	0.484 **	0.899 **	−0.095 *–0.381 **
Consequences	7. My NPC has major consequences on my life	0.648	0.714	0.571 **	0.767 **	−0.079 *–0.442 **
	8. My NPC does not have much effect on my life	0.745	0.733	0.319 **	0.588 **	−0.231 **–0.282**
	9. My NPC strongly affects the way others see me	0.690	0.718	0.523 **	0.687 **	−0.067–0.419 **
	10. My NPC has serious financial consequences	0.692	0.720	0.498 **	0.691 **	−0.243 **–0.361 **
	11. My NPC causes difficulties for those who are close to me	0.657	0.716	0.555 **	0.749 **	−0.214 **–0.394 **
Personal Control	12. There is a lot I can do to control my symptoms	0.753	0.742	0.150 **	0.716 **	−0.102 *–0.337 **
	13. What I do can determine whether my NPC gets better or worse	0.670	0.735	0.260 **	0.814 **	−0.089 *–0.384 **
	14. The course of my NPC depends on me	0.700	0.735	0.272 **	0.779 **	−0.092 *–0.331 **
	16. I have the power to influence my NPC	0.723	0.744	0.125 **	0.762 **	−0.162 **–0.377 **
Treatment Control	20. My treatment will be effective in curing my NPC	0.705	0.743	0.080 *	0.866 **	−0.233 **–0.395 **
	21. The negative effects of my NPC can be prevented (avoided) by my treatment	0.769	0.740	0.152 **	0.835 **	−0.176 **–0.407 **
	22. My treatment can control my NPC	0.741	0.739	0.168 **	0.851 **	−0.167 **–0.387 **
Illness Coherence	24. The symptoms of my condition are puzzling to me	0.774	0.760	−0.164 **	0.729 **	−0.366 **–0.003
	25. My NPC is a mystery to me	0.723	0.759	−0.129 **	0.836 **	−0.382 **–−0.003
	26. I don’t understand my NPC	0.702	0.757	−0.105 **	0.875 **	−0.377 **–0.069
	27. My NPC doesn’t make any sense to me	0.727	0.754	−0.044	0.828 **	−0.318 **–0.043
	28. I have a clear picture or understanding of my condition	0.865	0.734	0.276 **	0.456 **	−0.051–0.268 **
Timeline Cyclical	29. The symptoms of my NPC change a great deal from day to day	0.868	0.728	0.380 **	0.735 **	−0.181 **–0.278 **
	30. My symptoms come and go in cycles	0.775	0.722	0.474 **	0.868 **	−0.303 **–0.439 **
	31. My NPC is very unpredictable	0.784	0.722	0.481 **	0.857 **	−0.383 **–0.469 **
	32. I go through cycles in which my NPC gets better and worse.	0.786	0.720	0.508 **	0.853 **	−0.208 **–0.559 **
Emotional Representation	33. I get depressed when I think about my NPC	0.902	0.710	0.635 **	0.859 **	−0.358 **–0.518 **
	34. When I think about my NPC, I get upset	0.884	0.711	0.615 **	0.914 **	−0.383 **–0.508 **
	35. My NPC makes me feel angry	0.910	0.717	0.544 **	0.827 **	−0.366 **–0.416 **
	37. Having this NPC makes me feel anxious	0.898	0.712	0.610 **	0.871 **	−0.291 **–0.469 **
	38. My NPC makes me feel afraid	0.900	0.712	0.612 **	0.864 **	−0.323 **–0.478 **

* *p* < 0.05, ** *p* < 0.01. Item-total correlation: Pearson correlation coefficient between each item and the overall scale. Item-subscale correlation: Pearson correlation coefficient between each item and its corresponding subscale. Item-other-subscale correlation: Pearson correlation coefficient between each item and the other subscale.

**Table 5:** Pearson correlation of the R-NPC-IPQ dimensions.

Subscale	Item Number	Range	Mean	SD	1	2	3	4	5	6	7
Subscale 1: Timeline (acute/chronic)	3	3–15	7.82	3.03	1						
Subscale 2: Consequences	5	5–25	15.20	3.89	0.403 **	1					
Subscale 3: Personal control	4	4–20	13.02	2.99	−0.099 *	−0.037	1				
Subscale 4: Treatment control items	3	3–15	11.07	2.13	−0.226 **	−0.140 **	0.466 **	1			
Subscale 5: Illness coherence items	5	5–25	15.77	3.77	−0.183 **	−0.287 **	0.070	0.096 *	1		
Subscale 6: Timeline cyclical	4	4–20	9.93	3.24	0.279 **	0.358 **	−0.156 **	−0.146 **	−0.371 **	1	
Subscale 7: Emotional representations	5	5–25	13.95	4.58	0.302 **	0.545 **	−0.136 **	−0.123 **	−0.397 **	0.526 **	1

* *p* < 0.05, ** *p* < 0.01. Mean, simple arithmetic mean, describes the centralized trend of item scores; SD, standard deviation, describes the dispersion trend of item scores.

**Table 6:** Pearson correlation among the R-NPC-IPQ dimensions and other related variables.

Illness Perception Questionnaire Subscale	Depression	Anxiety
Subscale 1: Timeline (acute/chronic)	0.224 **	0.166 **
Subscale 2: Consequences	0.415 **	0.338 **
Subscale 3: Personal control	−0.122 **	−0.134 **
Subscale 4: Treatment control	−0.135 **	−0.148 **
Subscale 5: Illness coherence	−0.261 **	−0.213 **
Subscale 6: Timeline cyclical	0.366 **	0.284 **
Subscale 7: Emotional representations	0.497 **	0.465 **

** *p* < 0.01.

## Data Availability

Data cannot be shared publicly because of the privacy implication, but is available upon resonable request. Data requests may be sent to the corresponding author.
